# FAVOR 2.0: A reengineered functional annotation of variants online resource for interpreting genomic variation

**DOI:** 10.1093/nar/gkaf1217

**Published:** 2025-12-03

**Authors:** Hufeng Zhou, Vineet Verma, Xihao Li, Zilin Li, Nicole Shedd, Thomas Cheng Li, Haoyu Yang, Alvin Zhang, Beatrice Borsari, Steven Buyske, Mark Gerstein, Tara Matise, Michael C Zody, Benjamin Neale, Zhiping Weng, Shamil R Sunyaev, Xihong Lin

**Affiliations:** Department of Biostatistics, Harvard T.H. Chan School of Public Health, Boston, MA 02115, United States; Department of Biostatistics, Harvard T.H. Chan School of Public Health, Boston, MA 02115, United States; Department of Biostatistics and Department of Genetics, University of North Carolina at Chapel Hill, Chapel Hill, NC 27599, United States; School of Mathematics and Statistics, Northeast Normal University, Changchun, Jilin 130024, China; Department of Genomics and Computational Biology, University of Massachusetts Chan Medical School, Worcester, MA 01605, United States; Department of Biostatistics, Harvard T.H. Chan School of Public Health, Boston, MA 02115, United States; Weston High School, Weston, MA 02493, United States; Department of Biostatistics, Harvard T.H. Chan School of Public Health, Boston, MA 02115, United States; Department of Biostatistics, Harvard T.H. Chan School of Public Health, Boston, MA 02115, United States; Middlesex School, Concord, MA 01742, United States; Program in Computational Biology and Bioinformatics, Yale University, New Haven, CT 06511, United States; Department of Molecular Biophysics and Biochemistry, Yale University, New Haven, CT 06520, United States; Department of Statistics, Rutgers, The State University of New Jersey, Piscataway, NJ 08854, United States; Program in Computational Biology and Bioinformatics, Yale University, New Haven, CT 06511, United States; Department of Molecular Biophysics and Biochemistry, Yale University, New Haven, CT 06520, United States; Department of Genetics, Rutgers, The State University of New Jersey, Piscataway, NJ 08854, United States; New York Genome Center, New York, NY 10013, United States; Program in Medical and Population Genetics, Broad Institute of MIT and Harvard, Cambridge, MA 02142 , United States; Department of Medicine, Massachusetts General Hospital, Boston, MA 02114, United States; Analytic and Translational Genetics Unit, Massachusetts General Hospital, Boston, MA 02114, United States; Department of Genomics and Computational Biology, University of Massachusetts Chan Medical School, Worcester, MA 01605, United States; Program in Medical and Population Genetics, Broad Institute of MIT and Harvard, Cambridge, MA 02142 , United States; Department of Biomedical Informatics, Harvard Medical School, Boston, MA 02115, United States; Department of Biostatistics, Harvard T.H. Chan School of Public Health, Boston, MA 02115, United States; Program in Medical and Population Genetics, Broad Institute of MIT and Harvard, Cambridge, MA 02142 , United States; Department of Statistics, Harvard University, Cambridge, MA 02138, United States

## Abstract

The Functional Annotation of Variants Online Resource (FAVOR), http://favor.genohub.org, is a whole genome variant annotation database and portal that provides comprehensive variant functional annotations of all possible variants across the genome. It can facilitate the analysis of whole-genome sequencing studies, support the interpretation of variant functional impacts, and help prioritize causal variants of diseases or traits. To support the growing popularity and expand the scope of FAVOR, we present here a substantial platform update. The new release features dramatically expanded annotations, a completely redesigned infrastructure powered by a newly implemented application programming interface (FAVOR-API), and a revamped web interface with advanced data-visualization capabilities and enhanced query performance. Key expansions include much more comprehensive variant annotations, including global, tissue- and cell-type–specific variant annotations; gene and protein annotations; support for both hg38 and hg19 reference genomes; and an interactive genome-browser for visualization of multi-faceted variant annotations. The updated platform also includes FAVOR-GPT, a large language model-powered interface for navigating the FAVOR database and interpreting results. FAVOR continues to evolve to keep pace with advances in research on interpreting the functional and phenotypic impact of genomic variation.

## Graphical Abstract

**Graphical Abstract:**
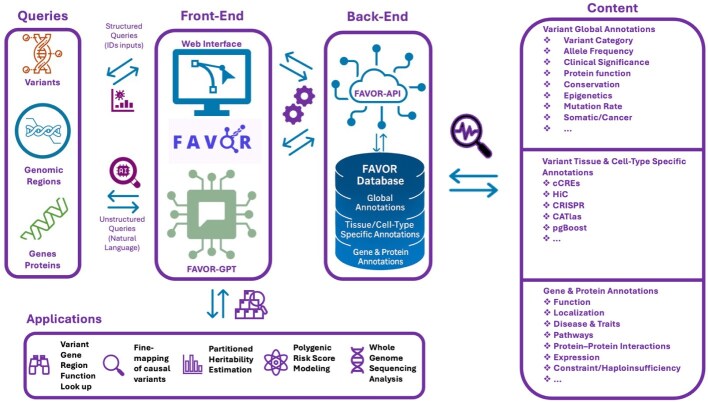

## Introduction

Functional annotation is essential for understanding the impact of genomic variation, as well as analyzing and interpreting genome-wide association studies (GWAS) and whole-genome sequencing (WGS) studies. GWASs have identified tens of thousands of variants associated with common human diseases and traits. Several large-scale WGS studies have emerged in recent years, including the Trans-Omics for Precision Medicine (TOPMed) program of the National Heart, Lung, and Blood Institute (NHLBI) [1], the Genome Sequencing Program (GSP) of the National Human Genome Research Institute (NHGRI) [2], the UK Biobank [3], and the All of Us Research Program [4]. These large WGS studies have identified over a billion coding and non-coding genetic variants across the human genome from more than a million individuals, enabling investigation of variants’ effects on common diseases and traits.

Functional Annotation of Variants Online Resource (FAVOR) [5, 6] was developed in 2023 to facilitate the analysis of WGS studies, support the interpretation of variant functional impacts, and help prioritize causal variants of diseases or traits. It is a whole-genome variant annotation database and portal with multi-faceted variant functional annotations of all possible 9 billion SNVs and observed INDELs across the genome. Since its launch, FAVOR has rapidly gained popularity in the genetic and genomic research community, with over 28 000 database downloads and over 69 000 portal annual visits. Despite its utility, the previous FAVOR resource has several limitations. It includes only global variant annotations based on the hg38 reference genome; lacks gene- and protein-level annotations; does not provide tissue- or cell-type–specific functional annotations; offers only limited visualization capabilities and interactive query features on its web interface; and does not support annotations based on the hg19 reference genome.

**Figure 1.**
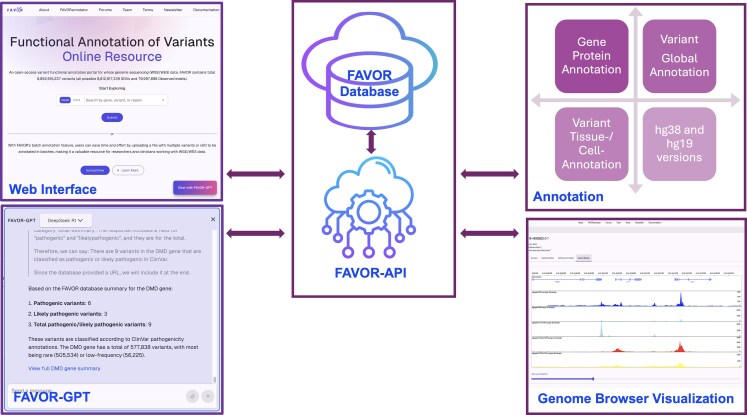
Overview of the updated FAVOR. The updated FAVOR has several major enhancements: (i) a revamped web interface that is more interactive and user-friendly; (ii) FAVOR-GPT, a generative natural language interface of FAVOR; (iii) substantially expanded annotations, including variant, gene, and protein annotations; enriched global variant annotations; tissue- and cell type–specific variant annotations; support for both hg38 and hg19; (iv) an interactive genome browser for visualization of multi-faceted variant annotations. The new API-enabled platform underpins these updates, ensuring improved performance and integration.

To address these limitations and enhance the practical utility of FAVOR for advancing genomic science, we have fully redesigned FAVOR by substantially expanding the FAVOR database and overhauling the FAVOR web interface (Fig. 1). The new FAVOR database includes (i) comprehensive gene and protein annotations; (ii) a significantly expanded collection of global variant functional annotations; (iii) tissue- and cell-type–specific variant functional annotations; and (iv) support for both hg38 and hg19 reference genomes. The revamped FAVOR web interface is built upon a fundamentally redesigned infrastructure powered by a newly implemented FAVOR-API. Key features of the new FAVOR portal include: (i) FAVOR-GPT [5, 6], an interactive large language model (LLM) interface that allows users to conveniently navigate and query the FAVOR database; (ii) an interactive, user-friendly web interface; (iii) an interactive genome browser capable of displaying multiple selectable variant functional annotations within a user-defined, zoomable region; and (iv) visualization modules for protein–protein interactions (PPIs) and pathway interaction networks. For example, the FAVOR interactive genome browser can display variants, genomic loci, tissue- and cell-type–specific regulatory elements and scores, and the linkage of variants to regulatory elements to genes with multi-layer functional annotations in any user-specified region.

FAVOR now runs on a cost‐effective institutional cloud platform to ensure long-term sustainability while retaining the scalable computing and storage required for large-scale whole-genome annotation. The entire web interface has been rewritten in Next.js to deliver instant, responsive browsing of variant and region‐level and gene and protein annotations on both desktop and mobile devices. The newly developed FAVOR-API also enables programmatic access for other standalone pipelines or web tools.

## FAVOR database updates

**Figure 2.**
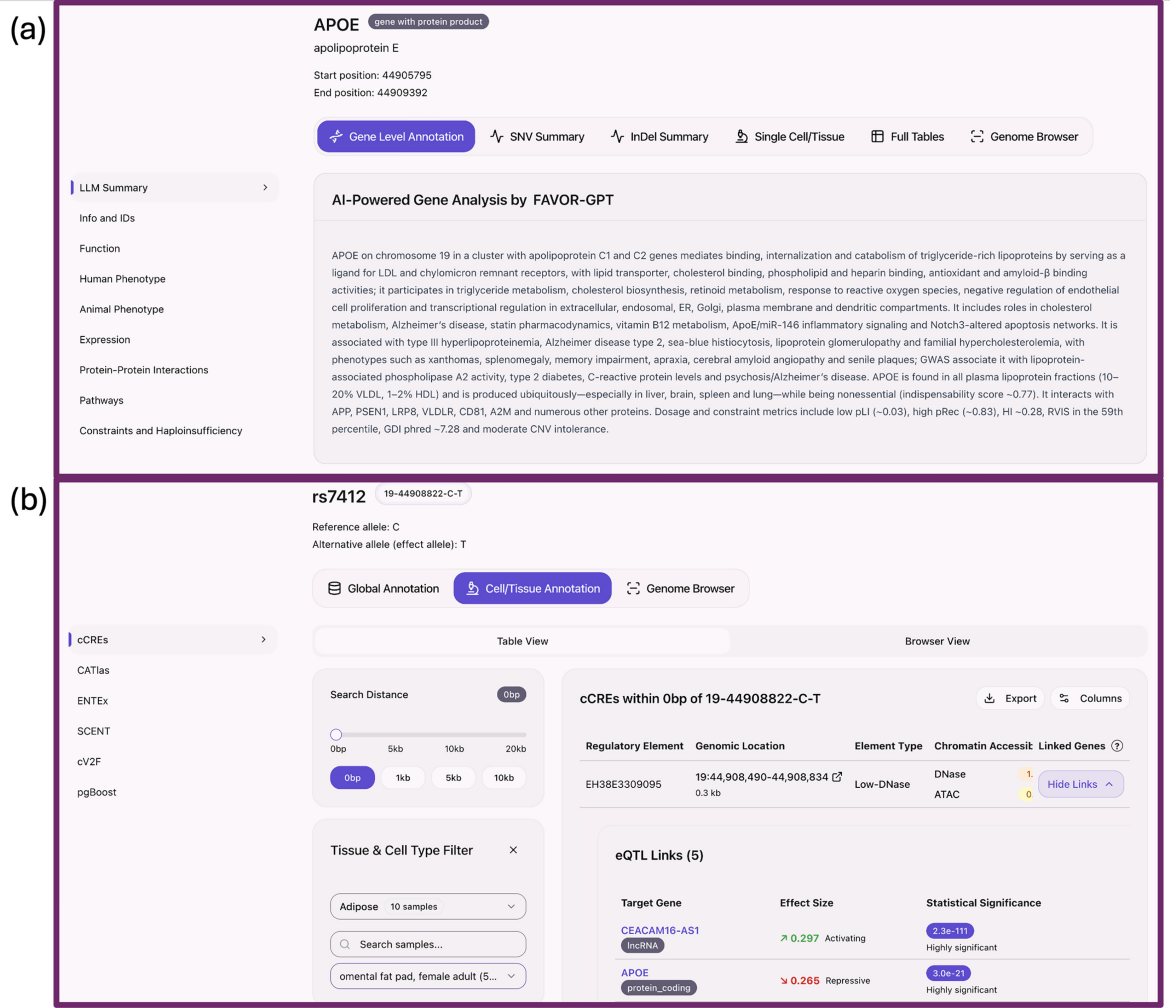
Illustration of the updated FAVOR annotations: (**a**) gene/protein level annotations and summary statistics of observed variants of a gene or a region; (**b**) variant tissue- and cell-specific multi-faceted variant functional annotations.

This new release of the FAVOR database provides far more comprehensive functional annotations of variants, genes, and proteins across the genome. The update introduces both gene- and protein-level functional annotations, adds and expands many new global variant functional annotations, expands coverage into tissue- and cell-type–specific functional annotations, and supports both hg19 and hg38 genome builds. Together, these advances transform FAVOR into a multi-omics and multi-resolution resource with broad utility in a wide range of applications, including functional annotation, genomic analysis, interpretation of variant impact, rare variant association analysis, fine-mapping, prioritization of causal variants of diseases or traits, and exploration of regulatory genomics (Fig. 2). Major annotation sources such as gnomAD, ClinVar, and GTEx will be updated at least annually or as new versions are released. Updates are documented on the FAVOR portal to ensure transparency and reproducibility.

### Gene- and protein-level functional annotations

The updated FAVOR database contains an expanded collection of gene- and protein-level annotations, functional descriptions, cross-references of multiple gene and protein databases with their accessions, impact on human and animal phenotypes, gene pathways and PPI networks, tissue-specific gene expression profiles, and constraint metrics. These additions strengthen FAVOR’s capabilities as a framework for interpreting the multi-faceted functional and phenotypic impact of genes and proteins. Since genomic variants often exert their impacts through genes and proteins, annotating the function, structure, and regulatory context of genes and proteins improves the interpretability of variant annotations.

Summarizing the different sets of identifiers and metadata for each gene is critical for coordinated reference. FAVOR now includes standardized gene symbols, synonyms, chromosomal coordinates, and identifiers from major databases such as Ensembl [7], RefSeq [8], UniProt [9], CCDS [10], and HGNC [11]. It also provides cross-references to rare diseases, genetics, and phenotype databases such as Orphanet [12], OMIM [13, 14], MGI [15, 16], and RGD [17, 18]. For each gene and protein, FAVOR includes curated functional descriptions and molecular roles, such as protein class [19], subcellular localization, HPA protein evidence [19], and pathway annotations from KEGG [20, 21], Wikipathways [22, 23], Biocyc [24, 25], ConsensusPathDB [26], BioCarta [27], and IntPath [28]. FAVOR also includes PPI networks, including BioGRID [29], IntAct [30], and HuRI [31].

These annotations help characterize gene and protein involvement in biological processes, such as DNA damage response, cell cycle checkpoints, ubiquitin-mediated proteolysis, and transcriptional regulation. To allow users to assess tissue specificity and expression contexts, FAVOR also provides gene expression profiles across diverse tissues from GTEx v8 [32]. Additional gene- and protein-level annotation metrics include mutation-intolerance and constraint scores (e.g. pLI [33], RVIS[34]), mutational-burden measurements (e.g. GDI [35]), and haploinsufficiency and constraints [36, 37]. Together, these features improve the biological interpretability of variants and their effects on genes and proteins (Fig. 2).

### Updates of global functional annotation scores

FAVOR variant-level global functional annotations have undergone a systematic update of refreshed conventional annotation scores and a significant expansion of newly added measurements. The previous version of the FAVOR global annotations includes the classical global measures, such as allele frequencies, protein functions, variant effect predictors, and evolutionary conservation. FAVOR now includes a broader range of emerging global variant annotations, including missense effect predictions (AlphaMissense) [38], genome‐wide mutation rate models (Mutation Rate) [39], deep learning splice prediction scores (SpliceAI) [40], somatic mutation annotations (COSMIC) [41], variant-phenotype associations (GWAS Catalog) [42], the latest allele frequencies from updated gnomAD v4 [43], updated ClinVar annotations [44], non-coding variants constrain scores like Gnocchi [43] and JARVIS [45], and new integrative scores such as MACIE [46]. By unifying these multifaceted data streams into a single platform, FAVOR provides comprehensive global functional annotations, offering users a one-stop resource to navigate, interpret, and prioritize variants for downstream study.

### Expansion into tissue-specific and cell-type–specific functional annotations

To support tissue- and cell-type-specific variant impact interpretation, FAVOR integrates a broad range of tissue- and cell-type–specific functional annotations. They include enhancer-target gene mappings (SCENT) [47] that leverage single-cell RNA- and ATAC-seq data, and pgBoost [47] SNP–gene links trained on combination of single-cell multi-omics peak–gene linking scores and genomic distance-based features on fine-mapped eQTL data. We have also enriched FAVOR with a diverse suite of epigenomic and regulatory maps, including EpiMap [48], the ENCODE candidate *cis*‐regulatory element (cCRE) registry [49], the single‐cell chromatin‐accessibility atlas CATlas [50], comprehensive MPRA data from high-throughput measurements [51, 52], genome-wide CRISPR screens [49, 53], and extensive eQTL datasets [54]. Together, these annotation resources provide insights from chromatin functional categories and enhancer activities through cell-type–specific accessibility and perturbation effects to gene expression changes, illuminating variant impacts in a multi-omics and multi-resolution functional landscape (Fig. 2).

### Supporting both hg38 and hg19 genome builds

Another development is FAVOR now provides comprehensive support for both hg38 and hg19 genome builds, enabling consistent variant annotation across legacy and modern datasets without reliance on coordinate conversion tools. A significant number of publicly available variant datasets, legacy GWAS results, and clinical variant reports remain mapped to hg19 coordinates. So this feature vastly expands FAVOR’s use, as individual variant queries and region-based analyses often rely on hg19 positions. The methodology used in the development of the FAVOR hg19 version resembles the technique used for the hg38 version. Users can now input variants mapped to either hg38 or hg19 directly into the main search interface, and FAVOR retrieves the corresponding annotations from the appropriate matching genome build.

To avoid possible inconsistencies from relying on coordinate conversion tools such as UCSC’s liftover [55], we elected to build the hg19 version database from the ground up. This approach ensures that all point-based annotations (e.g. allele frequencies, variant protein function scores) are derived natively from hg19 genomic positions, rather than approximated through liftover. The computation of annotation principal components (aPCs) [56] in hg19 version, which summarize multi-dimensional functional scores into interpretable metrics, such as aPC-protein function, aPC-conservation, aPC-epigenetics, is calculated in the same way as in the hg38 version and demonstrates a high degree of concordance. The hg19 version significantly broadens the utility of FAVOR and ensures compatibility with a wider range of legacy datasets.

## New FAVOR web interface

The revamped FAVOR web interface is more user friendly, highly interactive, and incorporates several new features that significantly enhance the user experience. Built with a responsive Next.js front end that communicates to the backend database via the high-performance FAVOR-API (Fig. 1), the platform now includes an interactive genome browser for visualizing multi-scale and multi-faceted functional impacts of genetic variations across the genome, visualization modules of PPI networks, and gene–gene interactions in pathways. We have also included FAVOR-GPT [5, 6], an interactive LLM chatbot for users to conveniently navigate and query the FAVOR database.

### FAVOR-API

The FAVOR-API is a lightweight, high-performance, scalable interface designed to provide streamlined access to the comprehensive variant-level, genomic region-level, and gene-/protein-level functional annotations in the backend database (Fig. 1). Developed with Go and deployed on the NERC OpenStack platform [57], the FAVOR-API supports rapid individual searches, FAVOR-GPT, and high-throughput batch retrievals and delivers structured JSON responses easily integrated into diverse analysis pipelines, software tools, and web applications. Besides variant, gene, and protein annotations, it also supports calculating gene- or region-based variant functional annotation summary statistics.

FAVOR-API supports the queries of up to five million variants per request, enabling efficient analysis of large inputs. It also supports scalable FAVOR online batch annotation with up to five million variants per upload. A Redis caching layer and carefully indexed PostgreSQL backend database ensure consistent millisecond-level response times, even under high demands. In addition to serving the web interface, the FAVOR-API also powers FAVOR-GPT, providing a unified channel that enables natural language queries, conventional queries through web interface, and functional annotation workflows.

### Revamped FAVOR web interface

By leveraging Next.js for server-side rendering and static generation, the redesigned FAVOR web interfaces are more user-friendly and interactive. It delivers fast page loads and fluid navigation of the FAVOR database on both desktop and mobile devices. FAVOR leverages the support of hybrid rendering to dynamically update annotation results while maintaining rapid load times for frequently accessed pages. Using Radix UI primitives, the design meets stringent accessibility standards and adapts to any screen size. Hosting on Vercel’s edge network guarantees automatic updates and low-latency responses to requests from all over the world. Beyond the interactive web interface, the online FAVOR batch annotation system has been redeveloped to robustly handle large uploads. It now features streaming compression and generates analysis-ready outputs with progress tracking and seamless downstream integration.

### FAVOR interactive visualization: genome browser and PPI and protein pathways interaction modules

**Figure 3.**
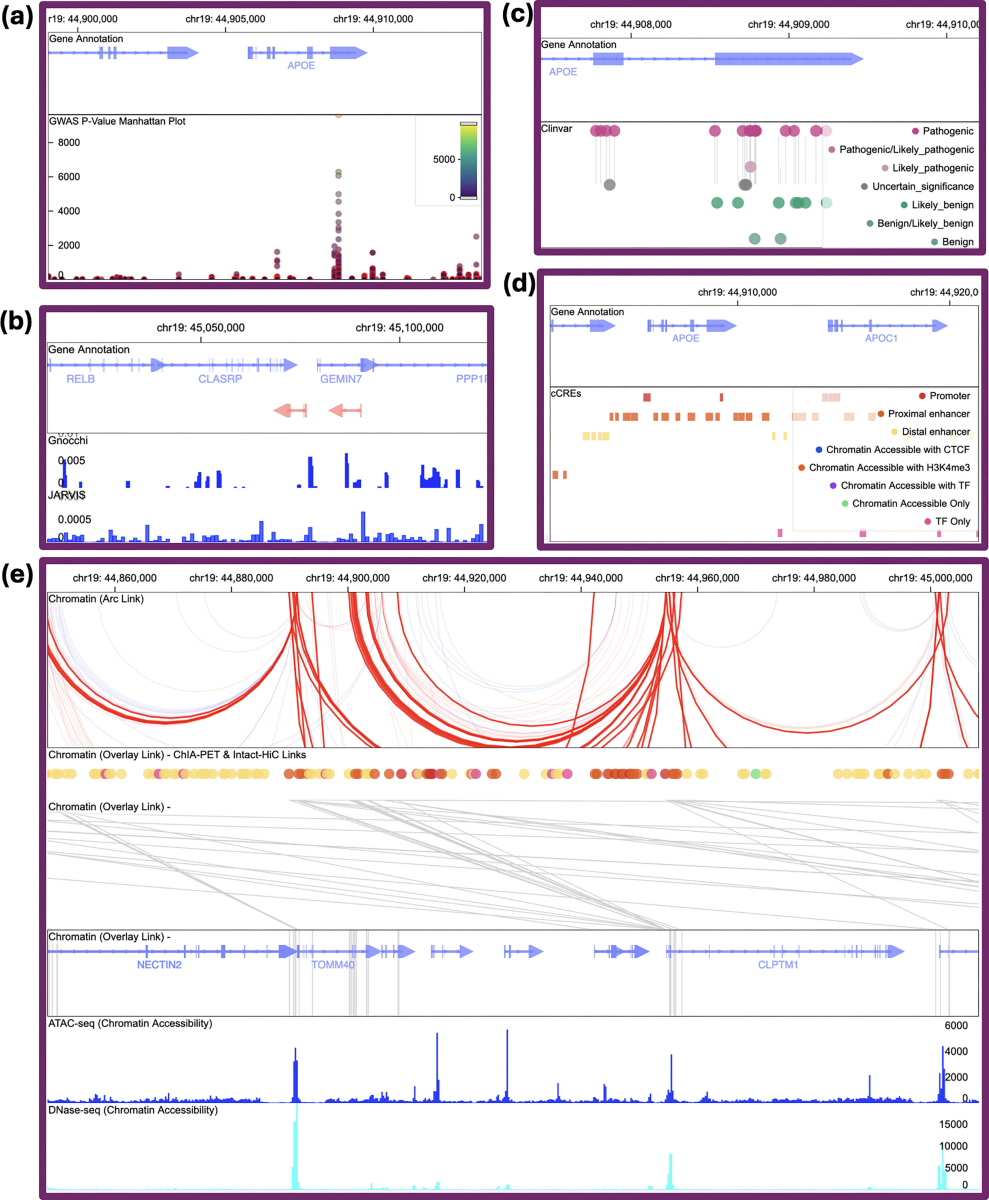
FAVOR Genome Browser visualization module: interactive exploration of multi-faceted impacts of multiple genetic variants within zoomable regions. (**a**) Variant annotations from the GWAS catalog; (**b**) Location-based variant constraint scores (Gnocchi and JARVIS); (**c**) Variant allele-specific annotation from ClinVar; (**d**) Global and tissue-specific and cell-type specific categorical annotation (cCREs) from ENCODE; (**e**) Chromatin conformation linkages of regulatory elements to genes shown in “Arc View” and “Overlay View” and coordinated visualization with continuous annotations of regulatory elements (DNase-seq and ATAC-seq).

**Figure 4.**
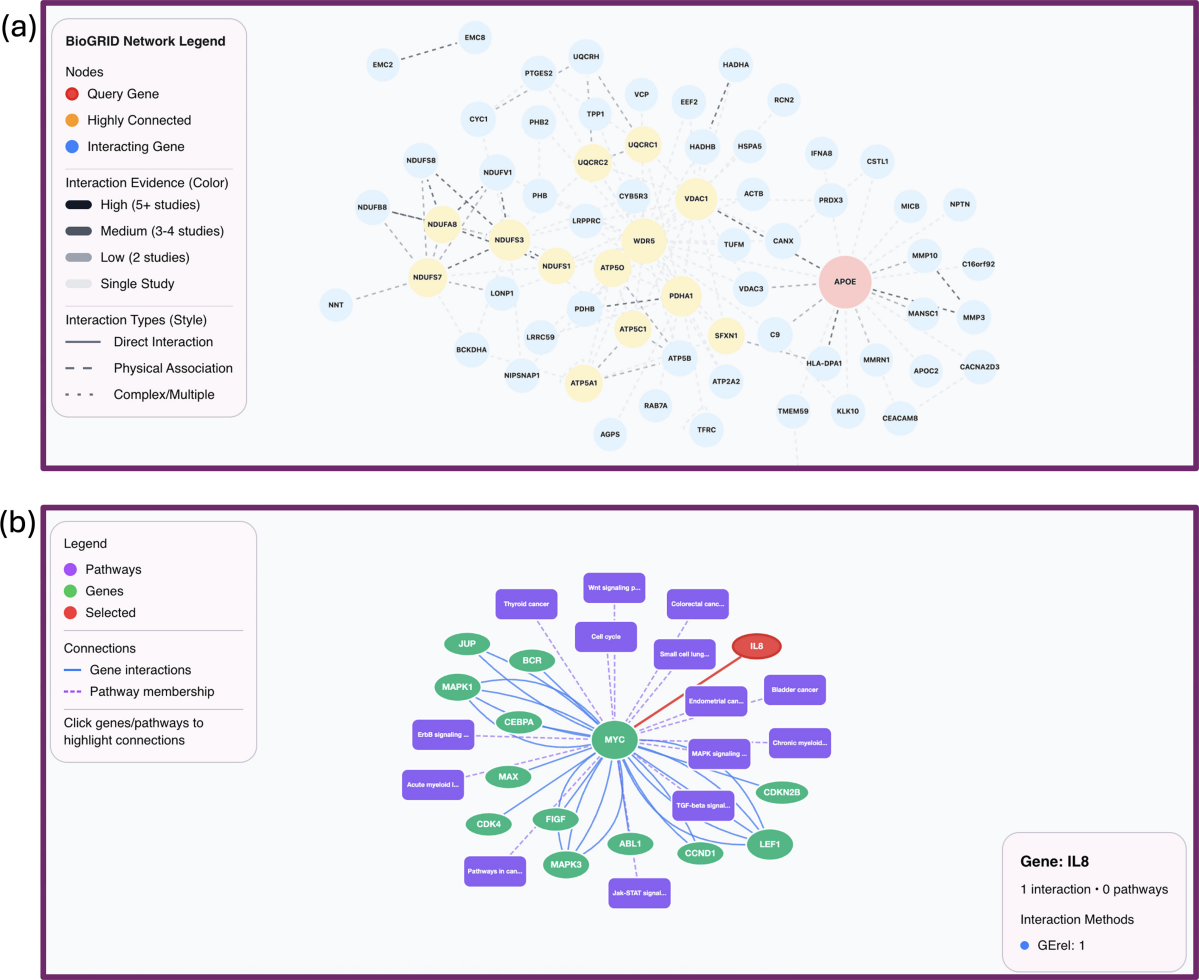
FAVOR network visualization module: (**a**) PPI networks, where users can select from three databases (BioGRID, IntAct, and HuRI), and (**b**) pathways, where users can select from four pathway databases (KEGG, BioCyc, WikiPathways, and IntPath). In both cases, users can specify the number of interactions to display.

The new release of the FAVOR portal provides an interactive genome browser visualization module for variants, genes, regulatory elements, and regulatory networks (Figs 3 and 4). It is built on Gosling [58] and HiGlass [59], and enables efficient rendering and interactive exploration of large multi-resolution genomic functional annotation data. The interactive genome browser allows users to explore multi-faceted variant functional annotations in a user-specified zoomable region through a dynamic, multi-layered interface that supports multiple data tracks (Fig. 3). For coding variants, one can visualize variant effect predictor categories, including those predicted by AlphaMissense, ClinVar categories, and conservation. For non-coding variants, one can visualize global (Gnocchi, JARVIS) and tissue- and cell-type–specific cCREs provided by ENCODE [49], as well as the linkage of variants in regulatory elements to genes. One can also visualize the variant-phenotype association GWAS results from the GWAS catalog [60].

Built upon the dynamic Cytoscape.js, the network visualization module enables interactive rendering of biological networks, such as PPIs, gene pathways, and regulatory networks (Fig. 4). Users can choose from different PPI databases, including BioGRID, IntAct, and HuRI, as well as different pathway databases, including KEGG [20, 21], Wikipathways [22, 23], Biocyc [24, 25] and IntPath [28]. Functional relationships are visually represented using color, shape, size, and edge style, enhancing the interpretability of large, information-rich networks. Users can manipulate layouts in real time, toggle annotation layers, and navigate from network nodes to detailed variant or gene pages without leaving the visualization (Fig. 4).

With the network and browser visualization modules, FAVOR serves as an intuitive and extensible platform that enhances integrative understanding of the relationships among variants, genes, and regulatory elements. For example, a user investigating a particular gene can immediately view its associated variants, associated regulatory elements, and its interactions with other genes and proteins in pathways. These modules smooth the processes of hypothesis generation, functional interpretation, and discovery of variant-to-gene, gene-to-pathway, and gene-to-gene relationships at scale.

### FAVOR-GPT: an LLM chatbot to query the FAVOR database

**Figure 5.**
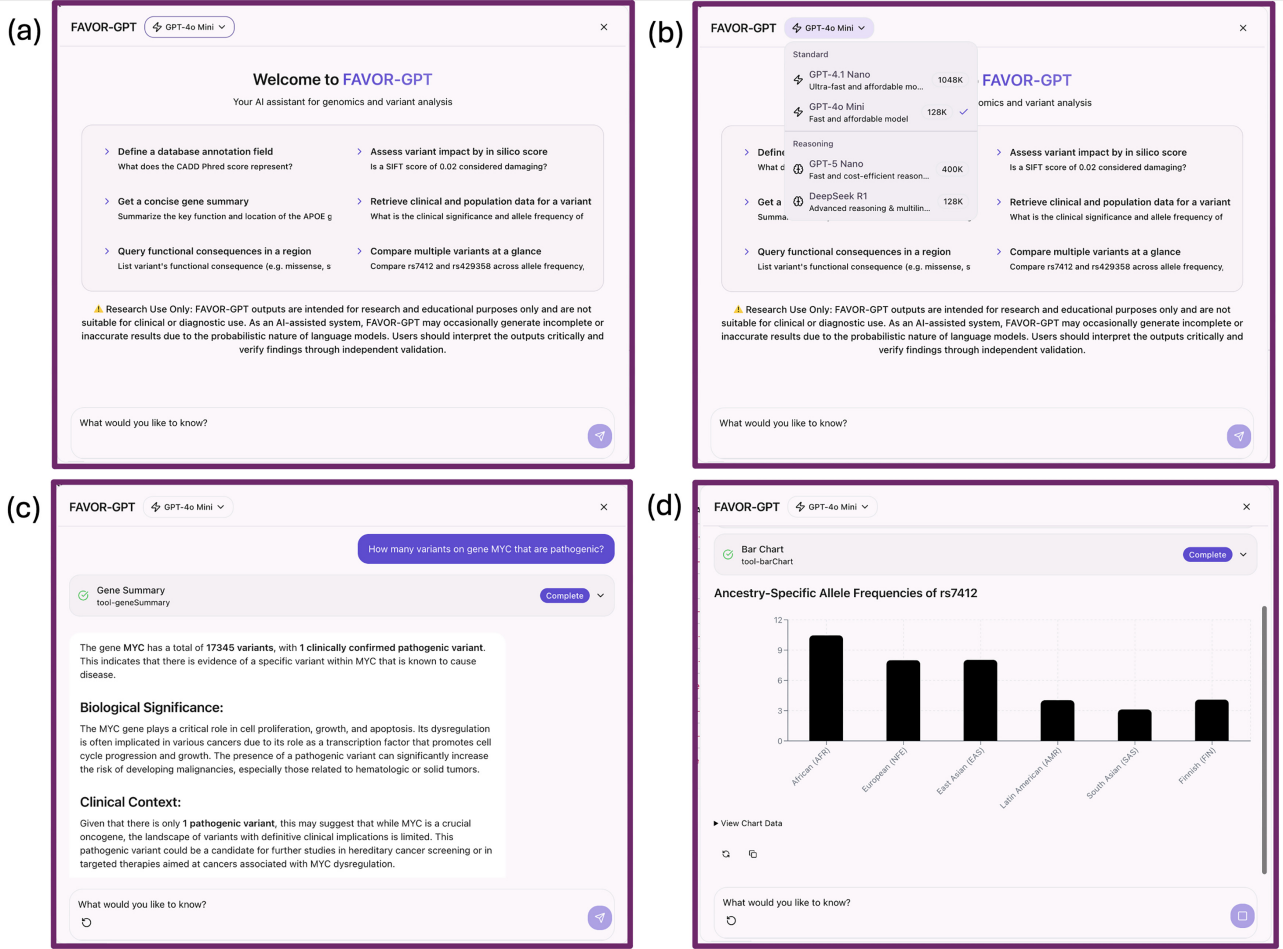
FAVOR-GPT: a natural language interface for variant function queries and summaries. (**a**) FAVOR-GPT interface; (**b**) option to select among different LLM models; (**c**) display of answering steps in response to a query; (**d**) in-dialog visualization of results, e.g. histograms.

We have built FAVOR-GPT [5], an interactive LLM tool that assists convenient interactive queries of the FAVOR database. FAVOR-GPT is built on the retrieval-augmented generation (RAG) framework by integrating several LLMs, including GPT 5 nano, GPT 4o min, GPT 4.1 nano, DeepSeek R1, with different reasoning capabilities, tailored to various analysis contexts and interpretive needs (Fig. 5). It is implemented in TypeScript using the Vercel.AI SDK and is embedded into the existing React/Next.js-based FAVOR web interface as a floating chat button. FAVOR-GPT enables real-time retrieval of variant functional annotations and provides interpretable, context-rich explanations of variants and genomic regions, as well as genes and proteins, while ensuring high fidelity of query results using the comprehensive genomic source data in the FAVOR database (Fig. 5).

To assess the reliability and accuracy of FAVOR-GPT, we systematically evaluated its responses by comparing the FAVOR-GPT outputs to manually verified annotations retrieved directly from the FAVOR web interface. In this validation, we confirmed that FAVOR-GPT’s RAG architecture consistently grounds all responses in the underlying FAVOR database. This design effectively minimizes the hallucinated or unsupported statements and ensures that generated summaries and interpretations remain faithful to the underlying genomic annotation data.

Setting an exemplar model for integrating AI-driven interfaces into genomic data resources, FAVOR-GPT has continued to evolve and improve since its original launch. The updated FAVOR-GPT fetches the expanded information in the FAVOR database, such as gene-/protein-level annotations, PPIs, pathways, and tissue- and cell-type–specific variant-level annotations, and phenotypic impact, through FAVOR-API, grounding LLM responses to natural language queries into accurate domain knowledge.

FAVOR-GPT also generates corresponding hyperlinks to the FAVOR pages for further reference, as well as dynamically calculating annotation summary statistics, e.g. the number of pathogenic variants, the number of loss-of-function variants, or the number of variants with minor allele frequencies between 1% and 5% in a gene or a region, and gene- or region-level variant distributions. To visualize these summary statistics, FAVOR-GPT creates in-dialog histograms, which extend usability of FAVOR (Fig. 5). Moreover, FAVOR-GPT supports multiple languages, breaking language barriers and making functional annotation queries accessible to non-English-speaking users and users with limited genetic backgrounds.

## Access and use for GWAS and WGS studies

The FAVOR portal and database [5, 6] have been widely used as revealed by the usage statistics (Supplementary Table S1). As a fully open-access resource, FAVOR database files are available in a compressed form on the Harvard Dataverse for efficient and effortless downloads. The FAVOR infrastructure supports flexible workflows, allowing users to annotate their variant datasets offline or deploy FAVORannotator on cloud platforms. FAVORannotator, which allows researchers to use the FAVOR database to functionally annotate any WGS and GWAS data, has been implemented on major cloud computing platforms, including the All of Us Researcher Workbench, UK Biobank Research Analysis Platform (UKBB-RAP), NHLBI TOPMed BioData Catalyst, and the Broad Institute’s Terra platform. In addition, FAVORannotator and FAVOR database have facilitated the deployment and widespread application of WGS rare variant analysis tools and software, such as STAAR [56], STAARpipeline [61], MetaSTAAR [62], MetaSTAARlite [63], and cellSTAAR [64]. The FAVOR database has been contributed to several data portals, including IGVF-catalog [52] and the Lipids Knowledge Portal [65].

## Discussion

The revamped and substantially expanded FAVOR database and portal represent a major re-envisioning of our original functional annotation platform, delivering a modern, scalable, and biologically enriched resource tailored for the analysis and result interpretation of a variety of genetic and genomic studies in both research and translational settings, such as GWAS and WGS studies. It also offers many more features compared to other existing functional annotation databases (Supplementary Table S2).

Looking ahead, the development roadmap for FAVOR includes continuing to expand and improve the quality and scope of the functional annotations, alongside continued enhancement of the user experience. In particular, we aim to advance variant, gene, and protein functional interpretation through integration of emerging experimental, genetic, genomic, and phenotypic data. Additionally, we plan to enable more comprehensive, content-aware exploration of variant functional landscapes by leveraging generative AI technologies to support advanced, analytic, and more user-friendly functional annotation navigation. We are also developing FAVOR-agent, an advanced evolution of FAVOR-GPT designed to further enhance AI-assisted functional annotation. FAVOR-agent will integrate multimodal data across genomic, transcriptomic, epigenomic, and proteomic layers, enabling a more comprehensive understanding of variant function within complex biological contexts. Beyond conversational data exploration, FAVOR-agent will also support natural language–driven data manipulation and workflow execution, allowing users to perform operations such as functional annotation of variant call format (VCF) files, genotype data (in VCF) to aGDS conversion, and integrative analysis entirely through intuitive natural language commands. By coupling new and lightweight LLMs with FAVOR’s curated knowledge base and scalable API, FAVOR-agent will extend the boundaries of interactive functional genomics and establish a new framework for automated, multimodal variant interpretation.

## Supplementary Material

gkaf1217_Supplemental_File

## Data Availability

The FAVOR portal is freely available at https://favor.genohub.org/ with detailed documentation. The FAVOR database can also be accessed at https://dataverse.harvard.edu/dataverse/favor. FAVOR-API can be found at https://docs.genohub.org/.
