# Supplementary material for: FAVOR 2.0: A reengineered functional annotation of variants online resource for interpreting genomic variation
Source: Nucleic Acids Res. 2025 Dec 3;54(D1):D1405–14. doi: 10.1093/nar/gkaf1217 (PMC12807660; doi:10.1093/nar/gkaf1217)
Supplement: gkaf1217_Supplemental_File [file gkaf1217_supplemental_file.docx]

**Supplementary Material**

**Supplementary Tables**

**Table S1**. Matrices of FAVOR impacts as of Sept 7^th^, 2025. This table records FAVOR impact matrices in multiple perspectives. (1) FAVOR web interface daily access counts from google analytics tracker; (2) FAVOR web interface weekly access counts from google analytics tracker; (3) FAVOR web interface yearly access counts from google analytics tracker; (4) FAVOR downloads counts record from Harvard Dataverse (combined counts for both full and essential database downloads); (5) FAVOR Publications (1,2) citations count; (6) FAVOR Publications (1,2) online views count; (7) Number of FAVORannotator apps on major cloud computing platforms e.g. UKBB-RAP, Terra, All of US research workbench, BioData Catalyst, Native Google Cloud, Native AWS Cloud, etc. (8) Number of pipelines and databases supported (analysis pipelines like STAAR(3), STAARpipeline (4), MetaSTAAR (5), MetaSTAARlite (6) and cellSTAAR (7) and data portals like IGVF-catalog (8,9) and IGVF Lipids Knowledge Portal (10), etc.).

| **Category** | **Matric** | **Value** |
| --- | --- | --- |
| **Web Access Tracker** | **FAVOR web interface access daily** | 1,586 |
|  | **FAVOR web interface access weekly** | 6,074 |
|  | **FAVOR web interface access yearly** | 69,031 |
| **Downloads** | **FAVOR Databases Downloads** | 28,419 |
| **Publications** | **Publications Citations** | 110 |
|  | **Publications Views** | 21893 |
| **Platform Deployments** | **FAVORannotator Apps on Cloud Platforms** | 6 |
| **Applications** | **Pipelines and Databases Supported** | 7 |

### Table S2  Comparison of different functional annotation resources: FAVOR (release 2025-09), CADD (11), dbNSFP(12), MyVariant(13), Varsome(14), RegulomeDB(15), VannoPortal(16).

| Resource | **FAVOR** | **CADD** | **dbNSFP** | **MyVariant** | **VarSome** | **RegulomeDB** | **VannoPortal** |
| --- | --- | --- | --- | --- | --- | --- | --- |
| **Variant coverage** | ≈9 B SNVs + select indels | ≈9 B SNVs + select indels | 81.5 M SNVs + 2.23 M splice SNVs | ≈1.45 B variants/documents | 33 B data points | Millions of regulatory sites | ≈9 B SNVs + select indels |
| **Interactive Genome Browser** | ✔ | ✖ | ✖ | ✖ | ✖ | ✖ | ✖ |
| **Gene and Protein Annotations** | ✔ | ✖ | ✖ | ✖ | ✖ | ✖ | ✖ |
| **Tissue-Cell-type specific Annotations** | ✔ | ✖ | ✖ | ✖ | ✖ | ✔ | ✔ |
| **Annotation principal components** | ✔ (aPC‑Protein, aPC‑Conservation, etc.) | ✖ | ✖ | ✖ | ✖ | ✖ | ✖ |
| **Natural‑language chatbot interface** | ✔ (FAVOR‑GPT) | ✖ | ✖ | ✖ | ✖ | ✖ | ✖ |
| **High‑throughput REST / CLI batch *(17)*** | ✔ (High performance REST and ≤100K variants / POST) | ▲ (CL files) | ✖ | ✔ (high‑perf REST) | ▲ (API, quotas) | ▲ Portal batch | ✖ |

✔ = fully supported; ▲ = partially / with limits; ✖ = not supported

Table S3. Comparison of differences between old and new FAVOR.

| **Functions and Features** | **Old FAVOR (version 2022)** | **New FAVOR (version 2025)** |
| --- | --- | --- |
| Tissue-specific functional annotation | ✖ | ✔ |
| Native support of HG19 build of annotation data | ✖ | ✔ |
| Gene-level functional annotation | ✖ | ✔ |
| Network visualization module | ✖ | ✔ |
| Genome Browser visualization module | ✖ | ✔ |
| FAVOR-API | ✖ | ✔ |
| FAVOR-GPT | ✖ | ✔ |
| Responsive web interface powered by React/Next.js | ✖ | ✔ |
| New hosting platform on private cloud | ✖ | ✔ |
| Discussion Forums for Q&A | ✖ | ✔ |
| Email notification service for updates | ✖ | ✔ |

✔ = fully supported; ▲ = partially / with limits; ✖ = not supported

Table S4. FAVOR-GPT query and answer examples.

| Queries | Model Used | Answers |
| --- | --- | --- |
| can you share the GENCODE category annotation of rs983056460? | GPT-4.1 Nano | The rs983056460 variant is categorized as "intergenic" according to the GENCODE comprehensive category annotation. This means it is located outside of gene coding regions and generally in a regulatory or non-coding part of the genome. Specifically, it resides near or within non-coding regions such as RN7SL318P (a non-coding RNA gene) and AC004944.1, at position 8:5,501,869 on chromosome 8. Biologically, intergenic variants can influence gene regulation by affecting enhancers, silencers, or other regulatory elements, although their direct impact often requires functional validation. Clinically, this particular intergenic location suggests a lower likelihood of directly altering protein function, but it may still be involved in gene regulation mechanisms affecting nearby genes or genomic regions. |
| how many SNVs with SIFT Deleterious annotation on MYC? | GPT-5 Nano | 71 SNVs in MYC are annotated as SIFT deleterious.  Interpretation:  These are SNV variants predicted by SIFT to be damaging to protein function. They are part of the overall SNV set in MYC and may contribute to altered function if they occur in coding regions.  Remember that in silico predictions (like SIFT) are one piece of evidence and should be integrated with other data (e.g., conservation, experimental validation) for clinical interpretation. |
| Create a population frequency visualization for rs7412 showing ancestry-specific allele frequencies | DeepSeek R1 | I'll create a population frequency visualization for rs7412 by first analyzing the variant data and then generating a bar chart. Biological and Clinical Context. rs7412 Functional Significance:  • This is a nonsynonymous SNV in the APOE gene (p.R176C amino acid change)  • Clinical classification: Drug response variant with pathogenic implications  • Associated conditions: Hypercholesterolemia, warfarin response, familial type 3 hyperlipoproteinemia  • Functional impact: Predicted as "probably damaging" by PolyPhen and "deleterious" by SIFT  Key Population Frequency Observations  The visualization reveals significant population stratification:  1. Highest frequencies in African populations (10.46%) - This suggests potential evolutionary selection or founder effects in African ancestry groups  2. Intermediate frequencies in East Asian (8.03%) and European (7.99%) populations - Consistent with global distribution patterns  3. Lowest frequencies in South Asian (3.11%) and Amish (1.11%) populations - These populations show the most restricted distribution  4. Notable variation: 10-fold difference between highest (African) and lowest (Amish) frequencies  Clinical Implications  This population stratification has important clinical consequences:  • Genetic testing interpretation: The same variant may have different clinical significance across populations due to varying allele frequencies  • Population-specific risk assessment: African ancestry individuals have higher baseline frequency, requiring adjusted risk calculations  • Pharmacogenomics: Warfarin and atorvastatin response predictions may need population-specific algorithms  The APOE ε2 allele (associated with rs7412) is known to have protective effects against Alzheimer's disease but increases risk for hyperlipidemia, creating complex clinical trade-offs that vary by population background. |
| List some variants in gene APOE with apc conservation score > 10 | DeepSeek R1 | APOE Variants with High Conservation Scores (APC > 10)  I found 10 variants in the APOE gene with APC conservation scores greater than 10, sorted by highest conservation first. Here's the biological interpretation of these findings:  Key Biological Insights:  1. High Conservation in Functional Regions  The top variants show exceptionally high conservation scores (27.5-23.7), indicating strong evolutionary constraint  This reflects APOE's critical role in lipid metabolism and Alzheimer's disease risk  2. Functional Impact Distribution  Exonic variants (protein-coding): 6 variants affecting amino acid changes  Upstream regulatory variants: 3 variants in promoter/enhancer regions  Intronic variant: 1 variant with potential splicing implications  3. Clinically Significant Variant  rs267606664 (position 44908730): Has clinical significance with "Uncertain_significance" classification but includes pathogenic associations for multiple conditions:  Alzheimer's disease (types 2, 3, 4)  Hypercholesterolemia  Lipoprotein disorders  Age-related macular degeneration  Notable Variants by Category:  High-Impact Exonic Variants:  rs770545391 (APC: 27.5): Nonsynonymous SNV predicted as deleterious by both SIFT and PolyPhen  rs988698841 (APC: 23.7): Nonsynonymous SNV with similar deleterious predictions  rs267606664 (APC: 22.4): Clinically significant variant with multiple disease associations  Regulatory Variants:  Upstream variants (positions 44905716-44905772) show strong GeneHancer enhancer connections to APOE and multiple zinc finger genes  These regulatory variants may affect APOE expression levels  Clinical Relevance:  The high conservation scores suggest these variants occur in functionally important regions of APOE. The presence of both coding and regulatory variants with strong conservation underscores APOE's critical role in:  Lipid metabolism and cardiovascular disease risk  Alzheimer's disease pathogenesis through amyloid-beta clearance  Immune function and inflammation regulation |
| How many Exonic variants (SNVs and INDELs) on gene MYC? | GPT-4o mini | Summary of Variants in MYC Gene  For the MYC gene, there are a total of variants classified as follows:  Exonic Variants (SNVs and INDELs):   - Total Exonic Variants: 622 - Single Nucleotide Variants (SNVs): 597 - Insertion/Deletion Variants (INDELs): 25   Biological Significance  The presence of these exonic variants in the MYC gene can have significant implications:  MYC Function: The MYC gene encodes a transcription factor that plays a crucial role in cell proliferation, growth, and differentiation. Variants in this gene can affect its function and may contribute to oncogenic processes.  Clinical Relevance:  Rare Variants: The vast majority of variants are classified as rare (859 out of 1294 total), suggesting that they may have less established roles in the general population, but may still be of clinical interest in specific contexts, especially in the study of cancer.  Pathogenic Potential: Despite the presence of variants, only one pathogenic variant has been identified, highlighting the importance of ongoing research to uncover the clinical implications of these variants.  Interpretation of Variants  Each exonic variant can differ in its clinical implications, with some potentially affecting protein function and therefore cellular processes, while others might have minimal impact. The study of these variants can contribute to understanding how MYC mutations relate to various cancers and guide therapeutic decisions. |
| How many variants in APOE with MAF >1% | GPT-4.1 nano | The FAVOR database indicates that the APOE gene contains a total of 1,470 variants. To focus on those with a minor allele frequency (MAF) greater than 1%, I consider variants classified as common, which in this dataset are variants with MAF > 1%.  According to the summary, APOE has 16 common variants with MAF > 1%.  Biological and clinical significance:  These common variants are typically tolerated in the population and may contribute to phenotypic diversity or disease susceptibility, depending on their functional impact.  APOE is well-known for its role in lipid metabolism and Alzheimer's disease risk, with specific alleles (e.g., ε2, ε3, ε4) associated with different disease risks.  The presence of 16 common variants suggests some variability in this gene within the population, some of which may have moderate effects or serve as markers in genetic studies. |

**Supplementary Reference**

1. Li, T.C., Zhou, H., Verma, V., Tang, X., Shao, Y., Van Buren, E., Weng, Z., Gerstein, M., Neale, B., Sunyaev, S.R. *et al.* (2024) FAVOR-GPT: a generative natural language interface to whole genome variant functional annotations. *Bioinform Adv*, **4**, vbae143.

2. Zhou, H., Arapoglou, T., Li, X., Li, Z., Zheng, X., Moore, J., Asok, A., Kumar, S., Blue, E.E., Buyske, S. *et al.* (2023) FAVOR: functional annotation of variants online resource and annotator for variation across the human genome. *Nucleic Acids Res*, **51**, D1300-D1311.

3. Li, X., Li, Z., Zhou, H., Gaynor, S.M., Liu, Y., Chen, H., Sun, R., Dey, R., Arnett, D.K., Aslibekyan, S. *et al.* (2020) Dynamic incorporation of multiple in silico functional annotations empowers rare variant association analysis of large whole-genome sequencing studies at scale. *Nat Genet*, **52**, 969-983.

4. Li, Z., Li, X., Zhou, H., Gaynor, S.M., Selvaraj, M.S., Arapoglou, T., Quick, C., Liu, Y., Chen, H., Sun, R. *et al.* (2022) A framework for detecting noncoding rare-variant associations of large-scale whole-genome sequencing studies. *Nat Methods*, **19**, 1599-1611.

5. Li, X., Chen, H., Selvaraj, M.S., Van Buren, E., Zhou, H., Wang, Y., Sun, R., McCaw, Z.R., Yu, Z., Jiang, M.Z. *et al.* (2025) A statistical framework for multi-trait rare variant analysis in large-scale whole-genome sequencing studies. *Nat Comput Sci*, **5**, 125-143.

6. Yohhan Kumarasinghe, J.W., Yuxin Yuan, Haoyu Zhang, Zilin Li, Xihao Li. (2025) MetaSTAARlite: An all-in-one tool for biobank-scale whole-genome sequencing meta-analysis. *medRxiv*, **2025.06.05.25328973**.

7. Buren, E.V., Zhang, Y., Li, X., Selvaraj, M.S., Li, Z., Zhou, H., Palmer, N.D., Arnett, D.K., Blangero, J., Boerwinkle, E. *et al.* (2025) cellSTAAR: Incorporating single-cell-sequencing-based functional data to boost power in rare variant association testing of non-coding regions. *bioRxiv*, 2025.2004.2023.650307.

8. IGVF. (2025), <https://catalog.igvf.org/>.

9. Consortium, I. (2024) Deciphering the impact of genomic variation on function. *Nature*, **633**, 47-57.

10. IGVF. <https://hugeamp.org:8000/research.html?pageid=igvf_lipids_front>.

11. Rentzsch, P., Witten, D., Cooper, G.M., Shendure, J. and Kircher, M. (2019) CADD: predicting the deleteriousness of variants throughout the human genome. *Nucleic Acids Res*, **47**, D886-D894.

12. Liu, X., Li, C., Mou, C., Dong, Y. and Tu, Y. (2020) dbNSFP v4: a comprehensive database of transcript-specific functional predictions and annotations for human nonsynonymous and splice-site SNVs. *Genome Med*, **12**, 103.

13. Chunlei Wu, A.S., Sean D. Mooney, et. al. . <https://github.com/biothings/myvariant.info>.

14. Kopanos, C., Tsiolkas, V., Kouris, A., Chapple, C.E., Albarca Aguilera, M., Meyer, R. and Massouras, A. (2019) VarSome: the human genomic variant search engine. *Bioinformatics*, **35**, 1978-1980.

15. Boyle, A.P., Hong, E.L., Hariharan, M., Cheng, Y., Schaub, M.A., Kasowski, M., Karczewski, K.J., Park, J., Hitz, B.C., Weng, S. *et al.* (2012) Annotation of functional variation in personal genomes using RegulomeDB. *Genome Res*, **22**, 1790-1797.

16. Huang, D., Zhou, Y., Yi, X., Fan, X., Wang, J., Yao, H., Sham, P.C., Hao, J., Chen, K. and Li, M.J. (2022) VannoPortal: multiscale functional annotation of human genetic variants for interrogating molecular mechanism of traits and diseases. *Nucleic Acids Res*, **50**, D1408-D1416.

17. (2022), <https://www.codementor.io/blog/batch-endpoints-6olbjay1hd>.
